# Relationship between gut microbiota and lymphocyte subsets in Chinese Han patients with spinal cord injury

**DOI:** 10.3389/fmicb.2022.986480

**Published:** 2022-09-26

**Authors:** Rizhao Pang, Junyu Wang, Yisong Xiong, Jiancheng Liu, Xin Ma, Xiang Gou, Xin He, Chao Cheng, Wenchun Wang, Jinqi Zheng, Mengyuan Sun, Xingang Bai, Ling Bai, Anren Zhang

**Affiliations:** ^1^Department of Rehabilitation Medicine, General Hospital of Western Theater Command, Chengdu, China; ^2^State Key Laboratory of Biotherapy, West China Hospital, Sichuan University, Chengdu, China; ^3^Department of Laboratory Medicine, General Hospital of Western Theater Command, Chengdu, China; ^4^Department of Rehabilitation Medicine, Shanghai Fourth People’s Hospital Affiliated to Tongji University School of Medicine, Shanghai, China

**Keywords:** spinal cord injury, gut microbiota, spinal cord injury induced immune depression syndrome, Chinese Han patients, lymphocyte subsets

## Abstract

This study is to investigate the changes of lymphocyte subsets and the gut microbiota in Chinese Han patients with spinal cord injury (SCI). We enrolled 23 patients with SCI and 21 healthy controls. Blood and fecal samples were collected. The proportion of lymphocyte subsets was detected by flow cytometry. 16S rDNA sequencing of the V4 region was used to analyze the gut microbiota. The changes of the gut microbiota were analyzed by bioinformatics. Correlation analysis between gut microbiota and lymphocyte subsets was performed. CD4 + cells, CD4 + /CD8 + ratio and CD4 + CD8 + cells in peripheral blood of SCI patients were significantly lower than those of the control group (*P* < 0.05). There was no significant difference in B cells and CIK cells between the SCI group and the control group. The gut microbiota community diversity index of SCI patients was significantly higher than that of healthy controls. In SCI patients, the relative abundance of Lachnospiraceae (related to lymphocyte subset regulation), Ruminococcaceae (closely related to central nervous system diseases), and Escherichia-Shigella (closely related to intestinal infections) increased significantly, while the butyrate producing bacteria (Fusobacterium) that were beneficial to the gut were dramatically decreased. Correlation analysis showed that the five bacterial genera of SCI patients, including Lachnospiraceae UCG-008, Lachnoclostridium 12, Tyzzerella 3, Eubacterium eligens group, and Rumencocciucg-002, were correlated with T lymphocyte subsets and NK cells. In the SCI group, the flora Prevotella 9, Lachnospiraceae NC2004 group, Veillonella, and Sutterella were positively correlated with B cells. However, Fusobacterium and Akkermansia were negatively correlated with B cells. Moreover, Roseburia and Ruminococcaceae UCG-003 were positively correlated with CIK cells. Our results suggest that the gut microbiota of patients with SCI is associated with lymphocyte subsets. Therefore, it is possible to improve immune dysregulation in SCI patients by modulating gut microbiota, which may serve as a new therapeutic method for SCI.

## Introduction

Patients with spinal cord injury (SCI) are prone to concurrent infection, which seriously affects their quality of life, and even threatens their life (Guillou, 1993). SCI can cause suppression of the non-specific and specific immune function, which is called spinal cord injury induced immune depression syndrome (SCIIDS) (Riegger et al., 2007). SCIIDS can increase the body’s sensitivity to infection and is one of the main causes of post-traumatic infection (Schnell et al., 1997; Held and Lane, 2014). SCIIDS is mainly manifested as abnormalities and functional impairment of T lymphocyte subsets and NK cells, which in turn affects the immune function of patients (Kliesch et al., 1996; Yang et al., 1999; Monahan et al., 2015). The immune system can be regulated by the sympathetic and parasympathetic nervous system *via* endocrine, hematopoietic, and immune organs such as spleen, bone marrow, and lymph nodes (Laginha et al., 2016). Thus, in SCI, the neural pathways that mediate immune responses may be disrupted. Because the central autonomic nerve pathway descends through the spinal cord, after SCI, the output signals from the preganglionic sympathetic axons responsible for innervating immune organs and adrenal glands are inhibited, resulting in immunosuppression (Lucin et al., 2007; Prüss et al., 2017). However, the underlying mechanism of SCIIDS remains unclear.

Recently, studies have found that the gut microbiota is closely related to immune function, and the disruption of the gut microbiota can lead to immune dysfunction (Round and Mazmanian, 2009; Hooper et al., 2012; Nicholson et al., 2012). The homeostasis of the gut microbiota plays an important role in maintaining the normal number of basophils and regulating the differentiation of T cells (Hill et al., 2012). The gut microbiota can stimulate CD4 + T cells to produce interferon and enhance the phagocytosis of monocyte-macrophages (Chen, 2013). Lactobacillus reuteri can induce the production of CD4 + CD8 + αα + double positive T cells in the intestinal epithelium. It is shown that the intake of Lactobacillus reuteri and tryptophan-rich foods can promote the reprogramming of intestinal epithelial CD4 + T cells into immune regulatory cells, thereby improving immune function (Cervantes-Barragan et al., 2017).

It has been reported that SCI can disrupt the balance of the gut microbiota (Gungor et al., 2016; Kigerl et al., 2016). Zhang et al. (2018) found that the diversity and structural composition of the gut microbiota in patients with SCI were reduced, indicating that the neurogenic rectum of patients with SCI is related to the gut microbiota. Kigerl et al. (2016) found that there was intestinal dysfunction and increased intestinal mucosal permeability after SCI, which in turn led to the translocation of gut microbiota. They believed that SCI-induced gut dysbiosis activated mucosal immune cells in gut-associated lymphoid tissue, which in turn affected intestinal permeability. However, there is no report on which gut microbiota is closely related to immune dysregulation after SCI.

In this study, we used 16S rDNA high-throughput sequencing and bioinformatics methods to analyze the changes of the gut microbiota in SCI patients. We detected changes in lymphocyte subsets in SCI patients and analyzed the relationship of the gut microbiota with lymphocyte subsets. Our findings may provide a new treatment alternative for improving the immune dysregulation in SCI patients by regulating the gut microbiota.

## Materials and methods

### Ethics statement

Prior written informed consent was obtained from every patient. This study was approved by the Ethics Committee of the Western Theater General Hospital of the Chinese People’s Liberation Army (Chengdu, China), and registered in the Chinese Clinical Trial Registration Center (Registration number: ChiCTR1800016823).

### Study cohort

Twenty-three SCI patients and 21 healthy controls (sex and age matched) were included in the study. Among the SCI patients, 18 were males and 5 were females, with an average age of (35.87 ± 8.275) years. In the controls, there were 16 males and 5 females with an average age of (37.05 ± 7.871) years. There was no statistically significant difference between the two groups in terms of sex and age (all *P >* 0.05). The clinical data of study cohort is shown in Tables 1, 2. All SCI patients were clearly diagnosed with SCI based on their injury history, clinical symptoms, CT examination, MRI (magnetic resonance) examination, etc. The degree of SCI was determined according to the “International Standards for Neurological Classification of SCI” revised by the American Spinal Injury Commission (Revised in 2019) (Wang J. et al., 2015). Healthy controls were recruited from the Physical Examination Center of The General Hospital of Western Theater Command.

**TABLE 1:** Baseline characteristics of SCI patients (*n* = 23) and healthy control (*n* = 21).

Categorical variables (SCI group)	N (%)	Categorical variables (Healthy control group)	N (%)
**Sex**		**Sex**	

Male	18 (78)	Male	16 (76)
Female	5 (22)	Female	5 (24)

**Age (year)**		**Age (year)**	

22–31	8 (35)	22–31	7 (33)
32–41	8 (35)	32–41	8 (38)
42–51	7 (30)	42–51	6 (29)

**Course of disease (month)**		**Course of disease (month)**	

1–50	13 (57)	/	/
51–100	6 (26)	/	/
101–200	3 (13)	/	/
201–300	1 (4)	/	/

**Damage plane**		**Damage plane**	

C_4_—C_8_	8 (35)	/	/
T_1_—T_6_	3 (13)	/	/
T_7_—T_12_	10 (43)	/	/
L_1_—L_5_	2 (9)	/	/

**ASIA impairment scale**		**ASIA impairment scale**	

Asia		Asia	
A	15 (65)	/	/
B	4 (17)	/	/
C	2 (9)	/	/
D	2 (9)	/	/

SCI, Spinal Cord Injury; ASIA, American Spinal Cord Injury Association.

**TABLE 2:** Blood and urine biochemical parameters of SCI patients (*n* = 23) and healthy control (*n* = 21).

Test	Normal range	SCI patients (average value)	SCI patients (range value)	Healthy controls (average value)	Healthy controls (range value)
White blood cell count	(3.5∼9.5) × 10^9^/L	6.36 × 10^9^/L	(3.98∼8.13) × 10^9^/L	6.18 × 10^9^/L	(3.53∼7.98) × 10^9^/L
Urinary leukocytes	0∼25/μL	24.5/μL	(3.3∼102.4)/μL	0.08/μL	(0∼1.2)/μL
Urinary bacteria	0∼1,000/μL	412.5/μL	(0.9∼1,214)/μL	0.27/μL	(0∼3.6)/μL

SCI, Spinal cord injury.

The inclusion criteria were: (1) Patients met the International Standard for the Neurological Classification of SCI (ASIA Standard); (2) The cause of SCI was trauma; (3) Patients were of Chinese Han nationality, aged between 18 and 60 years old; (4) Patients did not use antibiotics, probiotics, etc. in the previous month; (5) Patients were without serious primary diseases (autoimmune diseases, tumors, etc.), lung infection, bladder infection, respiratory tract infection, respiratory failure, heart failure or other serious complications; (6) Patients did not suffer from severe diarrhea, inflammatory bowel disease or other gastrointestinal diseases within a month; (7) Patients had no history of fecal bacteria transplantation; (8) Patient had no obvious mental disorders, depression, or anxiety.

### Blood and fecal sample collection

Fresh peripheral whole blood samples (2 ml each) collected from the middle cubital vein of each subject after fasting. The tests with blood samples were finished within 6 h. Fecal samples (0.5∼1 g of the central part of the fecal mass) were collected from all subjects and put them into sterile fecal collection tubes. The tube lids were closed immediately after sample collection to keep the samples in a relatively anaerobic environment as much as possible. The fecal samples were stored at –80°C and were not repeatedly frozen and thawed. During transportation, the fecal samples were placed in a dry refrigerator.

### Flow cytometry analysis

T lymphocyte subpopulations (including CD3 +, CD4 +, CD8 +, CD4– CD8–, and, CD4 + CD8 + cells), NK cells, B cells and CIK (cell cytokine induced killer) cells in peripheral blood were detected by using flow cytometry (BD FACSCantoer II). Briefly, the samples were incubated with corresponding antibodies at room temperature in the dark for 20–30 min. After hemolysis in at room temperature in the dark for 10 min, the samples were analyzed by flow cytometry.

### 16S rDNA sequencing

The PowerFecal™ DNA extraction kit (Qiagen, Germany) was used to extract DNA samples from fecal samples. The 16S rDNA V4 region of the samples were amplified. The PCR products of the same sample were mixed after PCR and then subjected to electrophoresis. Then the relative bands were recovered with gel recovery kit for 16S rDNA sequencing. The primers with Barcode were: 515F (5′-GTGCCAGCMGCCGC GGTAA-3′) and 806R (5′-GGACTACHVGGGTWT CTAAT-3′) (Caporaso et al., 2011; Liu et al., 2017). The sequencing was performed on the Illumina Hiseq sequencer platform (Illumina, USA).

### Bioinformatics analysis

For operational taxonomic units (OTU) analysis, UPARSE software (Edgar, 2013) (version 7.1)^1^ was used to classify all sequences. The similarity greater than 97% was clustered into one OTU, and the OTU was filtered by the latest version (SSU115) provided by the official website of uparse.

For diversity analysis, α-diversity was evaluated by species richness index (PD and Chao1) and species diversity index (Shannon and Simpson). Based on unweighted UniFrac algorithm, β diversity was generated and analyzed according to principal coordinate report (PCoA) and non-metric multidimensional scaling (NMDS).

For Taxonomy analysis, the Bayesian algorithm of nucleic acid database classification was used to perform taxonomic analysis on OTU representative sequences with a similarity level of 97%. A supervised comparison of the microbiota between the SCI and healthy groups was performed by utilizing the Linear discriminant analysis (LDA) effect size (LEfSe), which is often used to identify the presence and effect size of region-specific OTUs among different groups (Segata et al., 2011; Eren et al., 2013).

### Statistical analysis

All data were analyzed using SPSS19.0. The measurement data were tested for normality and homogeneity of variance with Kruskall-Wallis test. If there is normal distribution and homogeneity of variance, data were expressed as mean ± SD and compared with *t*-test; otherwise, data were expressed as median (interquartile range) and analyzed with the non-parametric test Mann-Whitney *U*-test. Chi square test is used for comparing counting data. Spearman correlation coefficient was used for correlation analysis. All graphs were plotted with Prism GraphPad 8 Software for Mac OSX and chiplot.^2^ The difference is statistically significant at *P* < 0.05.

## Results

### T lymphocyte subsets, NK cells, B cells and CIK cells in spinal cord injury patients and healthy controls

**FIGURE 1:**
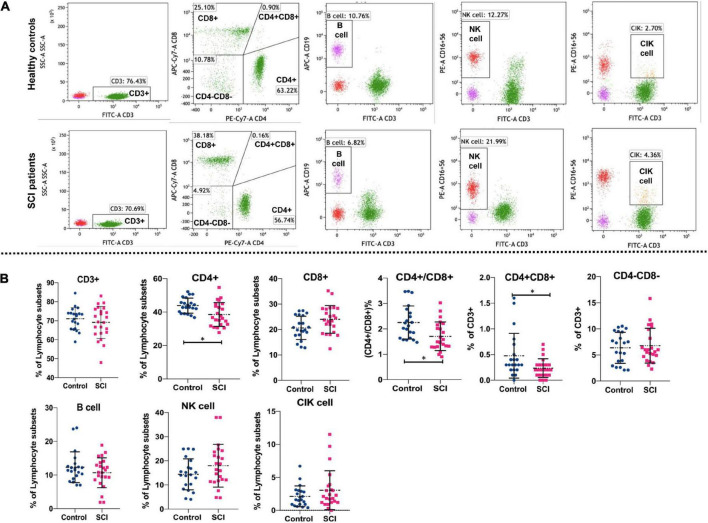
Comparison of lymphocyte subsets in SCI group and control group. Flow cytometry analyzed major T lymphocyte subsets, NK cells, B cells and CIK cells. Representative **(A)** and quantitative results **(B)** were shown. The cells analyzed included CD3 + cells, CD4 + cells, CD8 + cells, CD4+/CD8+, CD4 + CD8+, CD4-CD8- cells, B cells, NK cells, and CIK cells. *P < 0.05.

Flow cytometry was used to detect the proportions of lymphocyte subsets. The gating strategy of lymphocyte subsets is shown in Supplementary Figure 1. The representative flow cytometry results are shown in Figure 1A. Statistically, the proportion of CD4 + T cells and CD4 + CD8 + cells as well as the ratio of CD4 + /CD8 + were also significantly lower in SCI patients as compared to healthy individuals (*P* < 0.05) (Figure 1B). However, there were no significant differences between SCI patients and healthy individuals in the proportions of CD3 + cells, CD8 + T cells, CD4-CD8- cells, NK cells, B cells, and CIK cells (*P* > 0.05). These data indicate a disruption in homeostasis of the CD4 + T cell subset in patients with SCI.

### Analysis of α and β diversity of the gut microbiota

**FIGURE 2:**
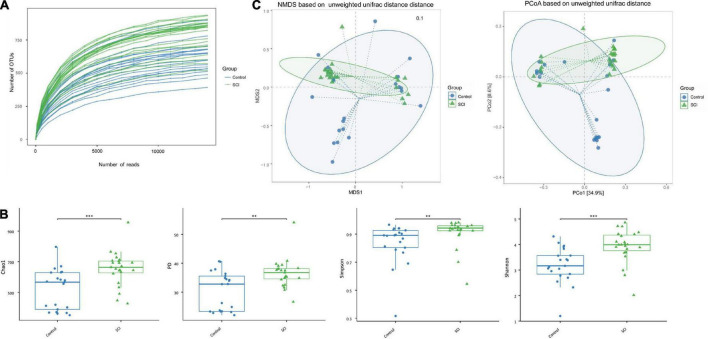
The a-diversity and b-diversity indexes of the gut microbiota in fecal samples of SCI patients and healthy controls. **(A)** Dilution curve of the samples. **(B)** The α-diversity was evaluated by species richness index (PD and Chao1) and species diversity index (Shannon and Simpson). Based on unweighted UniFrac algorithm. Each box represents the median, interquartile range, minimum and maximum values. **(C)** The β diversity was analyzed with NMDS and PCoA. **p < 0.005, ***p < 0.001.

After Illumina HiSeq sequencing, we obtained 1,495,203 valid sequences from 44 stool samples, and the average number of sequences per sample was 33981.89 ± 2671.78. The length was between 292 and 300 bp, the average length was 294.34 ± 1.98 bp, and the similarity was over 97%. We further used OTU clustering to compare all clean tags with OTU sequences and obtained 1,445,867 high-quality sequences. The average read of each sample was 32860.61 ± 2585.99, and the number of OTUs per sample was 26,775. Among them, there were 709,416 valid sequences in healthy controls, with an average sequence of 33781.71 ± 2381.93. After OTU clustering, 686,097 high-quality sequences were obtained (the average number of sequences was 32671.29 ± 2288.05). There were 785,787 valid sequences in patients with SCI, and the average number of sequences was 34164.65 ± 2899.29. After OTU clustering, 759,770 high-quality sequences were obtained (the average number of sequences was 33033.48 ± 2819.69). To further explore the trend of microbiota richness with sequencing depth, a sparse curve was plotted (Figure 2A), which indirectly reflects the species richness of the sample.

The average community diversity index of SCI patients (that is, the alpha diversity based on the OTU level, including Chao1 index, PD index, Simpson index, and Shannon index) was significantly higher than that of the healthy control group (Figure 2B), indicating that the degree and diversity of the gut microbiota in SCI patients are significantly higher than those of the healthy control group. In addition, there were significant differences in β-diversity between the SCI group and the healthy control group (Figure 2C). In order to reflect the difference in the two-dimensional coordinates of the samples, PCoA and NMDS were performed (Figure 2C). The analysis showed that the gut microbiota structure of SCI patients was different from that of control group. Diversity analysis found that there were significant differences in the gut microbiota structure between SCI patients and healthy subjects, which were consistent with the findings of increased gut microbiota diversity in central nervous system diseases such as Parkinson’s disease.

### Analysis of the composition of the gut microbiota at the phylum, family, and genus levels

**FIGURE 3:**
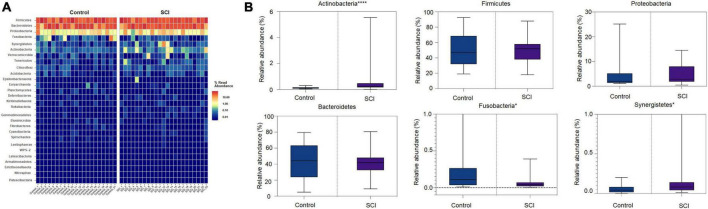
Analysis of the composition of the gut microbiota at the phylum level. **(A)** Heat map of the gut microbiota at the phylum level. **(B)** The relative abundance of dominant microbiota at the phylum level. Each boxplot represents the median, quartile range, minimum and maximum values. Each dot represents a subject. The data was expressed as median (interquartile range); *P < 0.05, ****P < 0.0001.

Heat map of the gut microbiota showed that compared with the control, the community structure of gut microbiota in SCI patients was similar in phyla, class, order, family, and genus, but the abundance of some communities was slightly different. The high-abundance species of the gut microbiota were displayed at the phylum, family, and genus level. We found that at the phylum level, Firmicutes, Bacteroides, and Proteobacteria had the highest relative abundance and were dominant phyla in the two groups (Figure 3A). Compared with healthy controls, the relative abundance of Fusobacteria in SCI patients was significantly reduced (*P* = 0.0315), and the relative abundance of Synergistetes (*P* = 0.0437) and Actinomycota (*P* < 0.0001) were significantly increased (Figure 3B). Among them, Actinobacteria was the landmark phylum of SCI group.

**FIGURE 4:**
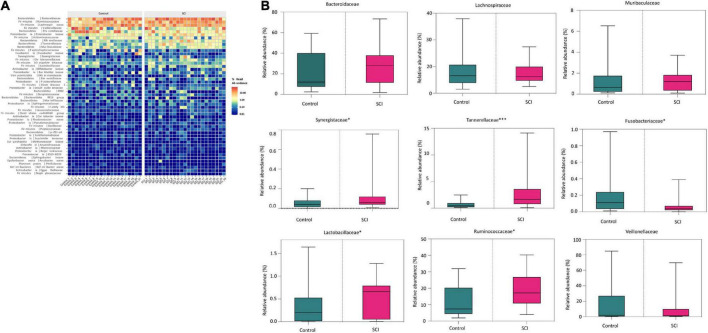
Analysis of the composition of the gut microbiota at the family level. **(A)** Heat map of the gut microbiota at the family level. **(B)** The relative abundance of dominant microbiota at the family level. Each box plot represents the median, interquartile range, minimum and maximum values. Each dot represents a subject. Data was expressed as median (interquartile range); *P < 0.05, ***P < 0.001.

At the family level, there were also differences in the composition of the gut microbiota between the SCI group and the healthy group (Figure 4A). In detail, the relative abundance of Tannerellaceae (*P* = 0.0006), Ruminococcaceae (*P* = 0.0265), Synergistaceae (*P* = 0.0488), Lactobacillaceae (*P* = 0.0442) etc. in SCI group were significantly higher while the relative abundance of Fusobacteriaceae (*P* = 0.0335) was significantly reduced than those in healthy control group (Figure 4B). Among them, Tannerellaceae were the iconic bacterial families of the SCI group.

**FIGURE 5:**
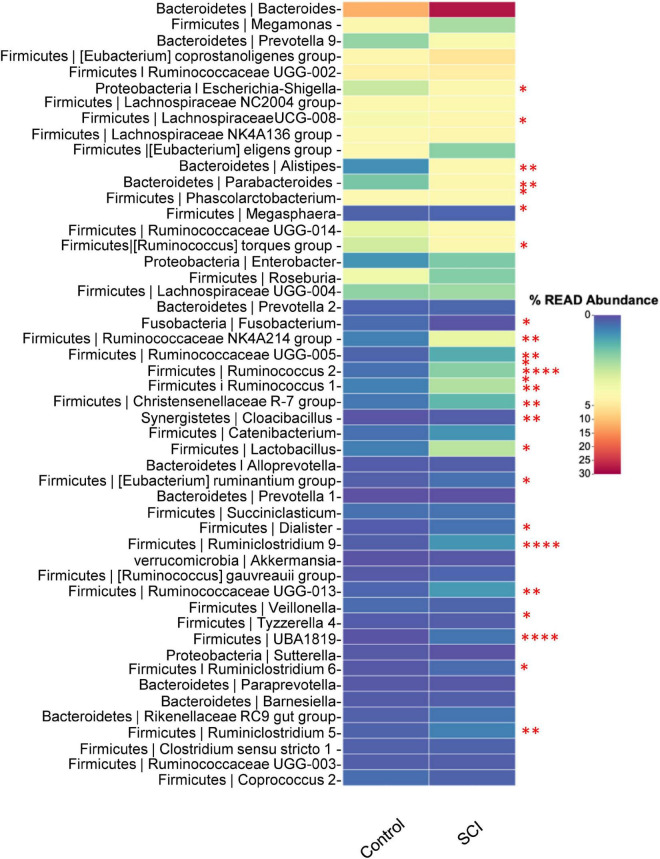
Analysis of the composition of the gut microbiota at the genus level. Heat map of microbiota in healthy subjects and patients with SCI. Data was expressed as median (interquartile range); *P < 0.05, ^**^P < 0.005, ^****^P < 0.0001.

At the genus level, the relatively abundant genera were displayed (Figure 5). Compared with the healthy control group, the average relative abundance of Escherichia-Shigella, LachnospiraceaeUCG-008, Alistipes, Parabacteroides, [Ruminococcus] torques group, Ruminococcaceae NK4A214 group, Ruminococcaceae UCG-005, Ruminococcus 2, Ruminococcus 1, Christensenellaceae R-7 group, Cloacibacillus, Lactobacillus, [Eubacterium] ruminantium group, Dialister, Ruminiclostridium 9, Ruminococcaceae UGG-013, UBA1819, Ruminiclostridium 6, Ruminiclostridium 5, etc. in the SCI group was significantly higher (*P* < 0.05), while the average relative abundance of Fusobacterium was significantly reduced (*P* < 0.05).

### Alteration in the taxa between spinal cord injury and healthy groups

**FIGURE 6:**
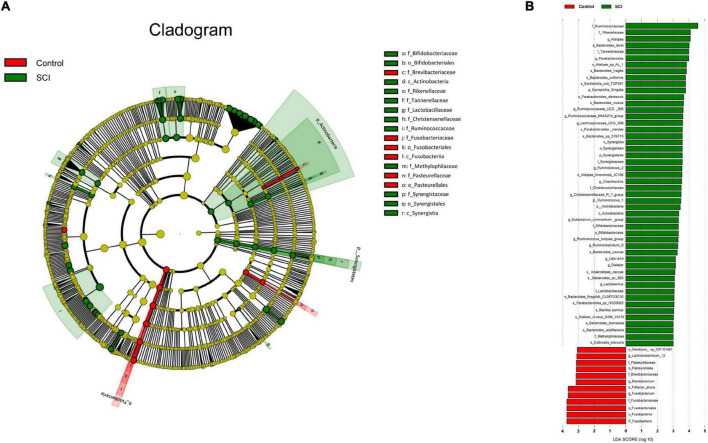
Taxonomic differences of gut microbiota in SCI and healthy groups. **(A)** Cladogram using LEfSe (Linear discriminant analysis effect size) method, which indicates the phylogenetic distribution of gut microbiota associated with SCI and healthy subjects. **(B)** LEfSe analysis revealed significant bacterial differences in gut microbiota between the SCI (positive score) and healthy groups (negative score). The LDA scores (log10) > 2 and *P* < 0.05 are listed.

We used a logarithmic LDA score cutoff of 2.0 to identify important taxonomic differences between the SCI and healthy groups. Our results suggested a remarkable difference in gut microbiota between the SCI and healthy groups based on LEfSe analysis. Here, we particularly considered differences in the taxa at the genus level. The relative abundances of the genera Fusobacterium, Brevibacterium and Lachnoclostridium_12 were higher in the healthy group than in the SCI group, whereas the relative abundances of genera Alistipes, Parabacteroides, Escherichia_Shigella, Ruminpcoccaceae_UCG_005, Ruminococcaceae_NK4A214_ group, Lachnospiraceae_UCG_008, Ruminococcus_2, Cloacibacillus, Christensenellaceae_R_7_group, Rumino coccus_1, Eubacterium_ruminantium _group, Rumino coccus_torques_group, Ruminiclostridium_9, UBA1819, Dialister and Lactobacillus were higher in the SCI patients than healthy controls (LDA score (log10) > 2, Figures 6A,B).

### Association between gut microbiota and clinical lymphocyte subsets in spinal cord injury patients and healthy controls

**FIGURE 7:**
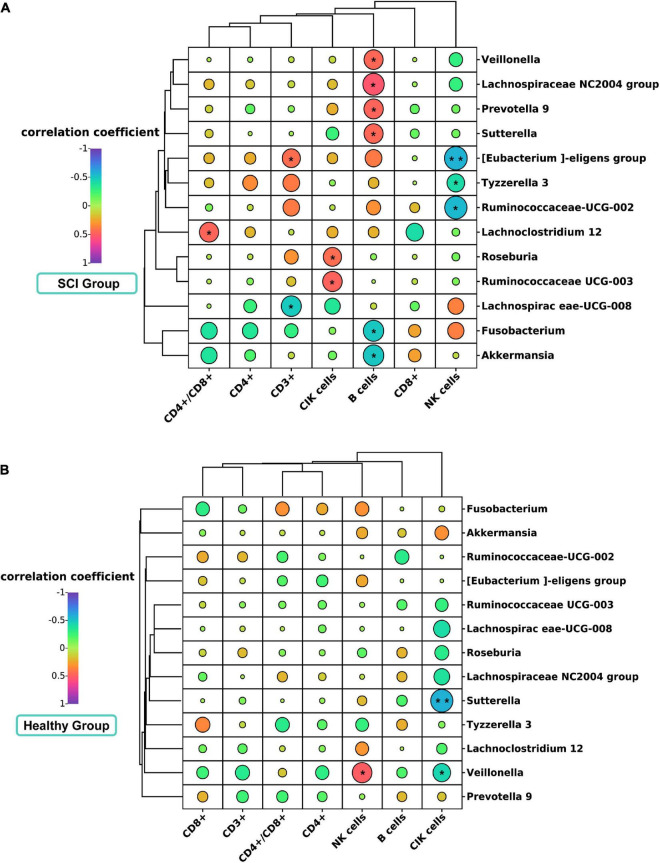
Correlation analysis diagram of microbiota and lymphocyte subsets in SCI patients and healthy groups. Correlation between gut microbiota (genus level) and immune cell population in SCI patients **(A)** and healthy groups **(B)**. Spearman’s rank correlation coefficient is indicated using a color gradient: red indicates positive correlation and blue indicates negative correlation. *P < 0.05; **P < 0.005.

We evaluated the relationship of gut microbiota (genus level and biomarker) with CD3+, CD4+, CD8+, CD4+\CD8+, B cells, NK cells, and CIK cells. In patients with SCI, Spearman correlation showed that Lachnospiraceae UCG-008 was significantly negatively correlated with CD3 + (Spearman rho = –0.487, *P* = 0.018, *q*-value > 0.05) (Figure 7A). Lachnoclostridium 12 was significantly positively correlated with the CD4+/CD8+ ratio (Spearman rho = 0.466, *P* = 0.025, *q*-value > 0.05). Tyzzerella 3 was significantly negatively correlated with NK cells (Spearman rho = –0.418, *P* = 0.047, *q*-value > 0.05). [Eubacterium] eligens group was significantly positively correlated with CD3 + (Spearman rho = 0.436, *P* = 0.037, *q*-value > 0.05), but was significantly negatively correlated with NK cells (Spearman rho = –0.573, *P* = 0.004, *q*-value > 0.05). Ruminiciccaceae UCG-002 was significantly negatively correlated with NK cells (Spearman rho = –0.553, *P* = 0.006, *q*-value > 0.05). Additionally, we further analyzed the correlation of B cells and CIK cells with gut microbiota (Figure 7A). Interestingly, we found that in the SCI group, the Prevotella 9 (Spearman rho = 0.471, *P* = 0.232, *q*-value > 0.05), Lachnospiraceae NC2004 group (Spearman rho = 0.532, *P* = 0.009, *q*-value > 0.05), Veillonella (Spearman rho = 0.4613, *P* = 0.0267, *q*-value > 0.05), and Sutterella (Spearman rho = 0.4612, *P* = 0.0268, *q*-value > 0.05) were positively correlated with B lymphocyte subsets, while Fusobacterium (Spearman rho = –0.499, *P* = 0.015, *q*-value > 0.05) and Akkermansia (Spearman rho = –0.504, *P* = 0.014, *q*-value > 0.05) were negatively correlated with B lymphocyte subsets. Meanwhile, we also found that Roseburia (Spearman rho = 0.440, *P* = 0.036, *q*-value > 0.05) and Ruminococcaceae UCG-003 (Spearman rho = 0.476, *P* = 0.022, *q*-value > 0.05) were positively correlated with CIK cell subsets. However, in the healthy control group, Veillonella (Spearman rho = 0.502, *P* = 0.020, *q*-value > 0.05) were positively correlated with NK cells, while Veillonella (Spearman rho = –0.441, *P* = 0.045, *q*-value > 0.05) and Sutterella (Spearman rho = –0.579, *P* = 0.006, *q*-value > 0.05) were negatively correlated with CIK cell subsets (Figure 7B). No other correlation was identified.

## Discussion

Studies have shown that the CD4 + /CD8 + ratio is significantly decreased in patients with some cancers (such as breast cancer, gastric cancer, and lung cancer) (Wang et al., 2013; Dias Rodrigues et al., 2017; Liu, 2022), HIV (Castilho et al., 2019), and neuromyelitis optica spectrum disorders (Yang et al., 2021), indicating that the immune function of patients may be reduced. The susceptibility of SCI patients to infection is related to immune disability, which is mainly manifested as disordered proportion of T cell subsets. Monahan et al. (2015) found that the number of CD4 + lymphocyte subsets decreased in patients with chronic SCI compared with healthy controls. Yang et al. (2003) found that the T cell immune function of patients with acute SCI was severely impaired, and the CD3 +, CD4 + and CD4/CD8 ratios were significantly lower than normal group. Consistently, our results showed that compared with healthy controls, the CD4 +, CD4 + /CD8 + ratio, and CD4 + CD8 + cells in patients with SCI were significantly reduced, while other T cell subsets, NK cells, B cells, and CIK cells were not statistically significant. At present, the mechanism of immune dysfunction in patients with SCI is currently unclear, which may be related to various factors such as impaired autonomic nerve function (Xu et al., 2004), post-traumatic stress disorder (Wang Z. et al., 2015), and psychological changes (Wang Z. et al., 2015). However, further studies are needed to further verify these findings.

At the phylum level, we observed a significant increase in the relative abundance of Actinobacteria and Synergistetes, etc. in SCI patients than in healthy controls. Meanwhile, we also found that the relative abundance of Fusobacteria in the SCI group decreased significantly. At the family level, we observed a significant increase in the relative abundance of the Ruminococcaceae of Firmicutes. In addition, we also found that the relative abundance of Tannerellaceae, Synergistaceae, Lactobacillaceae, etc. in SCI patients was higher than that of healthy controls. Meanwhile, we also found that the relative abundance of Fusobacteriaceae in the SCI group decreased significantly. At the genus level, we found that the relative abundance of Ruminococcaceae NK4A214 group, Ruminococcaceae UCG-005, Ruminococcus 2, Ruminococcus 1, Alistipes, Parabacteroides, | [Ruminococcus] torques group, Escherichia-Shigella, LachnospiraceaeUCG-008, Christensenellaceae R-7 group, Cloacibacillus, Lactobacillus, [Eubacterium] ruminantium group, Dialister, Ruminiclostridium 9, Ruminococcaceae UGG-013, UBA1819, Ruminiclostridium 6, Ruminiclostridium 5, etc. increased significantly in patients with SCI. Ruminococcus is closely related to central nervous system diseases and is negatively related to N-acetylaspartic acid, serum serotonin, and cortisol (a marker of nervous system health) (Mudd et al., 2017). Then, we found that Alistipes, Escherichia-Shigella, Ruminococcaceae UCG-005, LachnospiraceaeUCG-008, etc. were important marker bacteria in the SCI group, among which Alistipes was the most important. Zhao et al. (2018) found that the increase in the relative abundance of Alistipes was positively correlated with the increase in the content of long-chain saturated fatty acids in the colon. Although studies have pointed out that the increased abundance of Alistipes is harmful to the body, it may be beneficial to patients with SCI (de Meij et al., 2016, 2018; Sun et al., 2017). This may be a feedback regulation of the body, because in SCI patients, there is a lack of control of the gastrointestinal system by the central nervous system. Although the specific mechanism of Alistipes promoting colonic movement is not clear, it is very meaningful to further study the effect of this genus on the colon.

We also found that the relative abundance of other intestinal pathogens (Escherichia-Shigella) increased in patients with SCI. Escherichia-Shigella is a pathogen in the intestinal tract, which can cause acute rectal and colitis (Peng et al., 2010). Therefore, in the intestinal management of SCI patients, we should pay close attention to the abundance of Escherichia-Shigella, and actively prevent various infections, dysentery, diarrhea and other intestinal diseases.

In addition, we also found a significant decrease in Fusobacteria, which could produce butyrate metabolites. Butyric acid has positive effects on human health and can reduce the expression of pro-inflammatory factors, such as tumor necrosis factor α, interferon γ, and interleukin (IL-6 and IL-8) (Meijer et al., 2010; Fung et al., 2012) by inhibiting the activity of nuclear factor KB (Vinolo et al., 2011). It has been found that butyric acid has a powerful anti-inflammatory effect on macrophages (Park et al., 2005; Chen et al., 2007) and can inhibit the inflammatory response of the central nervous system (Kim et al., 2007). In terms of anti-cancer effects, butyric acid can induce cell differentiation and mediate cell apoptosis, thereby inhibiting cell proliferation and exerting its anti-cancer effects (Goncalves and Martel, 2013). In enhancing the defense function of the colon, butyric acid can enhance the main components of the intestinal mucus layer, and also participates in the regulation of the expression of a variety of mucus genes (Gaudier et al., 2004). Studies have shown that butyric acid can reduce food intake, improve insulin resistance, promote fat oxidation, and prevent obesity (Xu et al., 2009; Hill et al., 2014). Although we identified decrease of Fusobacteria in SCI patients, further study on the metabolome of microbiota is needed in the future to verify whether the production of butyric acid is affected by the metabolomics.

Lymphocytes play an important role in the host’s immunity, and their number indirectly reflects the immune status (Chen et al., 2017). There is a confirmed relationship between gut microbiota and lymphocyte subsets. Belotserkovsky et al. (2018) found that Shigella could target activated CD4 + T cells. Chen L. et al. (2018) found that specific diet could alter the intestinal microbiota of mice, leading to the proliferation of CD4 + T cells and promoting colitis caused by overexpression of IL23. Other studies have shown (Maslowski and Mackay, 2011; Chen B. et al., 2018; Linehan et al., 2018; Shenoy et al., 2019; Wu et al., 2019) that human-associated commensal bacteria have important regulatory effects on CD4 + T cells or CD8 + T cells.

It is found that children with Crohn’s disease had increased Enterobacteria and decreased Laospirillidae and Rumenococcus (Haberman et al., 2019). Saresella et al. (2017) found that a high-vegetable/low-protein diet increased the abundance of Lachnospiraceae in patients with multiple sclerosis. Lachnospiraceae is related to butyrate metabolism and is positively correlated with the number of anti-inflammatory immune cells. Consistently, we found a correlation between Lachnospiraceae and lymphocyte subsets. In SCI patients, Lachnospiraceae UCG-008 was negatively correlated with CD3 + T cells. Eubacterium eligens group had a significant positive correlation with CD3 + T cells, and a negative correlation with NK cells. Lachnoclostridium 12 and the CD4 + /CD8 + ratio were significantly positively correlated. Tyzzerella 3 is significantly negatively correlated with NK cells. Whether these four bacteria of Lachnospiraceae can interact with CD3 + T, CD4 + T, CD8 + T and NK cells is worthy of further study. We also found that Ruminococcaceae UCG-002 was negatively correlated with NK cells. Tanoue et al. (2019) isolated 11 strains of bacteria, including Ruminococcaceae, from healthy human feces and found that when they were co-colonized in the intestine, they effectively induced the proportion of CD8 + T cells in the mouse intestine and other organs. Additionally, they also enhanced the anti-tumor immunity mediated by interferon-γ + CD8 T cells. Although we found that Ruminococcaceae UCG-002 had a certain relationship with the NK cells of SCI patients, whether it directly participates in the proliferation and differentiation of NK cells needs further exploration.

Probiotics have been used to treat urinary tract infections and gastrointestinal discomfort in SCI patients. For example, Wong et al. (2014) found that taking live Lactobacillus casei (containing at least 6.5 × 10^9^CFU) for 7 days could reduce the incidence of antibiotic-associated diarrhea in SCI patients. Anukam et al. (2009) found that the Lactobacillus rhamnosus and Lactobacillus reuteri may help to down-regulate urinary tract infection-related inflammatory factors (TNF-α, IL-6, IL-8, IL-10 and IL-12) in patients with SCI. In an animal study, it was found that intervention with VSL#3 (a medical-grade probiotics) triggered a protective immune response in the gut-associated lymphoid tissue of SCI mice, activate Treg lymphocytes, improve immune function, and promote the recovery of motor function (Kigerl et al., 2016). In addition to probiotics intervention, the method of fecal microbiota transplantation (FMT) is also effective in improving motor function and anxiety-like behavior after SCI. Jing et al. (2021) showed that FMT could improve the motility and gastrointestinal function of SCI mice by regulating the gut microbiota, and the mechanism may be related to the increase of short- and medium-chain fatty acids. Moreover, FMT treatment can reduce the pathological damage, enhance the integrity of the blood-spinal cord barrier, inhibit the activation of microglia and astrocytes, and increase the secretion of neurotrophic factors, and ultimately promote the functional recovery of SCI mice (Jing et al., 2022). In addition, Schmidt et al. (2020) observed that FMT could improve the gut dysbiosis and reduce anxiety-like behaviors of SCI rats. Our findings provide further evidence for treating SCI by targeting gut microbiota.

It is reported that there is SCIIDS during SCI (Riegger et al., 2007), there is currently a lack of standard diagnostic criteria in clinical work. Based on previous findings (Beattie, 2004; Meisel et al., 2005; Popovich, 2014; Schwab et al., 2014) and our clinical experience, patients with SCI have decreased immunity, including immunosuppression, during acute phase, subacute phase, and even recovery periods, which is also the reason why patients are more likely to suffer infection during hospitalization. Therefore, the patients with SCI included in this study may all have SCIDS during the progression of their disease, but as their condition gradually recovers, their immune function may also recovers. It is very interesting to investigate the differences between SCI with/without SCIIDS and healthy controls. However, there is no standard clinical diagnostic criteria for SCIIDS. Moreover, due to the difficulty of patient recruitment and the drop out of patients with infection, only 23 patients with SCI were included in this study. This study only preliminarily analyzed the relationship of gut microbiota with T lymphocytes, NK cells and B cells. Our original intention was to attract more clinicians and researchers to pay attention to the relationship between the gut microbiota and immune function in patients with SCI. However, due to insufficient sample size and lack of SCIDS diagnostic criteria, the difference between SCI with SCIIDS and SCI group without SCIIDS cannot be further analyzed. We plan to conduct a multi-center clinical trial with larger sample sizes in the following work.

## Conclusion

In conclusion, changes in lymphocyte subsets in patients with SCI may be related to the disturbance of gut microbiota. In the future, it is possible to alleviate the state of immune dysregulation in patients with SCI through the regulation of gut microbiota, which may provide a new therapeutic method for SCI. However, the causal relationship between immune response and gut microbiota after SCI should be further clarified using fecal microbiota transplant model animals.

## Data availability statement

The raw sequencing data of this study have been deposited in the Genome Sequence Archive for human in Chinese National Center for Biotechnology Information (CNCB) (accession number HRA003029) (https://bigd.big.ac.cn/gsa-human/browse/HRA003029).

## Ethics statement

The studies involving human participants were reviewed and approved by the Ethics Committee of the Western Theater General Hospital of the Chinese People’s Liberation Army (Chengdu, China). The patients/participants provided their written informed consent to participate in this study.

## Author contributions

AZ, RP, and JW: study design. RP, JW, YX, JL, XM, XG, XH, CC, and LB: data collection. WW: data interpretation. XB: statistical analysis. RP and JW: manuscript preparation. JZ and MS: literature search. All authors have read and approved the manuscript.

## Footnotes

1
http://drive5.com/uparse

2
https://www.chiplot.online/

## Supplementary material

Supplementary Figure 1 Gating strategy of lymphocyte subsets. Flow cytometry dot plots illustrate the gating of B cells, CD3 + T cells, NK cells, and CIK cells. These cells were indicated by the expression of CD19+, CD3+, CD16+56+, and, CD3+CD16+56+, respectively. CD8+ T cells or CD4+ T cells were further gated on CD3 + T cells.
