# Supplementary figures and images for: Relationship between gut microbiota and lymphocyte subsets in Chinese Han patients with spinal cord injury
Source: Front Microbiol. 2022 Sep 26;13:986480. doi: 10.3389/fmicb.2022.986480 (PMC9549169; doi:10.3389/fmicb.2022.986480)

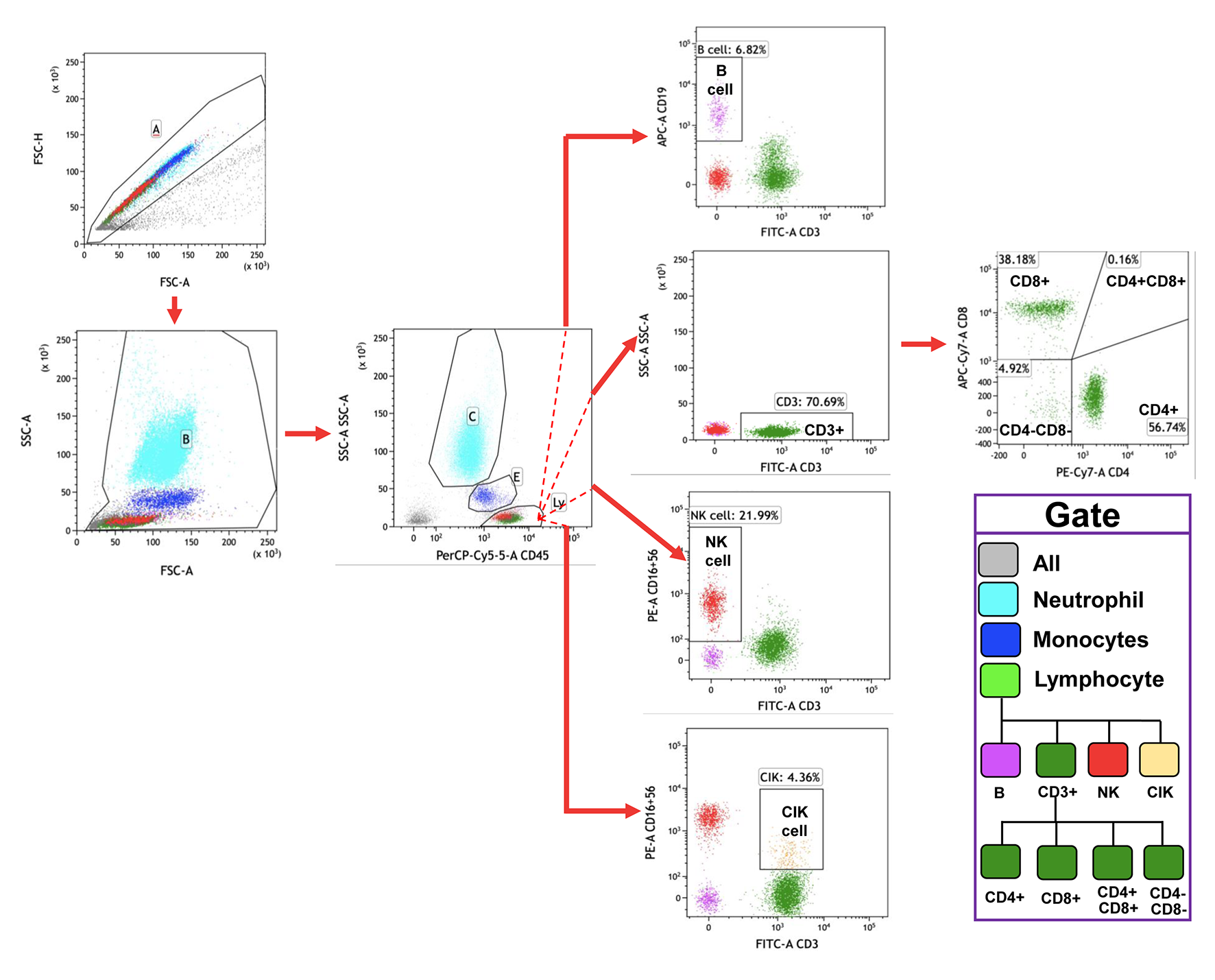

Supplement: Supplementary Figure 1 — Gating strategy of lymphocyte subsets. Flow cytometry dot plots illustrate the gating of B cells, CD3 + T cells, NK cells, and CIK cells. These cells were indicated by the expression of CD19+, CD3+, CD16+56+, and, CD3+CD16+56+, respectively. CD8+ T cells or CD4+ T cells were further gated on CD3 + T cells. [file Image_1.TIF]
